# Influence of Drying Methods on the Morphological Features, Microstructural Properties, and Antioxidant Performance of *Floccularia luteovirens*: A Metabolomic Analysis

**DOI:** 10.3390/jof11010078

**Published:** 2025-01-19

**Authors:** Mengjun Xiao, Tao Wang, Chuyu Tang, Min He, Xiaojian Pu, Tingjing Zhao, Yuling Li

**Affiliations:** State Key Laboratory of Plateau Ecology and Agriculture, Qinghai Academy of Animal and Veterinary Science, Xining 810016, China; 15574237597@163.com (M.X.); 13085500761@163.com (T.W.); chuyutang0410@163.com (C.T.); himi1228@163.com (M.H.); puxj@qhu.edu.cn (X.P.); 1996990038@qhu.edu.cn (T.Z.)

**Keywords:** *Floccularia luteovirens*, sensory evaluation, untargeted metabolomics, antioxidant activity

## Abstract

*Floccularia luteovirens* (*F. luteovirens*) has garnered increasing attention as an ingredient in both the pharmaceutical and food industries. Depending on the drying method, the accumulation of metabolites can greatly affect the quality. This research employed an untargeted metabolomics (LC-MS/MS) strategy to elucidate the similarities and differences in the morphological characteristics, microstructure, antioxidant capacity, and metabolic profiles of *F. luteovirens* subjected to three distinct drying methods: natural air-drying (YG), oven-drying (HG), and vacuum freeze-drying (DG). Our findings indicated that the color of *F. luteovirens* samples dried using the YG and HG methods was yellow-brown, exhibiting a high degree of browning, whereas the samples processed by the DG method displayed a golden-yellow hue and a desirable fullness. Regarding microstructure, the *F. luteovirens* samples from the YG and HG methods exhibited small and unevenly distributed pores, in contrast to the samples from the DG method, which were structurally intact and characterized by large inter-tissue pores. The antioxidant activity exhibited by *F. luteovirens* samples, which were processed using the DG method, was found to be significantly superior compared to the antioxidant activity of samples dried using two other methods. A correlation analysis indicated a significant link between antioxidant capacity and lipid as well as lipid-like molecules. Metabolomic analysis identified 1617 metabolites across 15 superclasses, with lipids, lipid-like molecules, organic acids and derivatives, and organic heterocyclic compounds being the predominant metabolites in *F. luteovirens*. Furthermore, KEGG enrichment analysis highlighted 20 pathways, indicating that the metabolism of amino acids could be significantly involved in the metabolic processes linked to the drying of *F. luteovirens*. This research clarifies how different drying techniques impact the metabolites or metabolic pathways of *F. luteovirens*, identifying the mechanisms that influence its quality and providing a reference for optimizing its processing and storage.

## 1. Introduction

Edible mushrooms encompass a varied collection of substantial fungi recognized for their distinct physiological characteristics and an extensive range of shapes [1,2]. They are celebrated for their delightful taste, rich nutritional profile, and an array of bioactive compounds, making them a popular choice for health-conscious consumers [3,4]. *Floccularia luteovirens*, an ectomycorrhizal fungus, is widely distributed across the Qinghai–Tibet Plateau [5]. Wild *F. luteovirens* from Qinghai is noted for its distinctive flavor and is abundant in proteins, polysaccharides, amino acids, and vitamins, exhibiting antioxidant and anti-tumor properties, thus establishing it as a valuable medicinal and edible fungus [6,7]. *F. luteovirens* is seasonal in its growth, and if not processed promptly after harvesting, it is prone to moisture loss, softening, rotting, and browning, which significantly diminish both its nutritional and commercial value [8]. Drying serves as a conventional technique for the preservation and processing of food [9]. At present, the frequently employed drying methods for edible mushrooms consist of natural sun-drying, hot air-drying, and vacuum freeze-drying [10,11,12]. The natural sun-drying method is straightforward, requires no equipment investment, is low-cost, and is not site-restricted; however, it is influenced by environmental conditions and has a prolonged drying time [13]. Hot air-drying involves blowing hot air into an oven or drying room to expedite the drying process. While this method is cost-effective, it can significantly damage heat-sensitive components [14]. In contrast, vacuum freeze-drying operates at low temperatures, resulting in minimal loss of volatile components in the material. This technique is particularly suitable for certain chemical products, pharmaceuticals, and food drying [15,16]. Various drying methods have been employed in the drying of *F. velutiper* [17], *Hericium erinaceus* [18], *Lentinus edodes* [19], *Pleurotus eryngii* [20], and *Volvariella volvacea* [21]. The influence of various drying techniques on different edible mushroom varieties differs markedly, affecting the sensory quality, nutritional components, and metabolites of the end products. Research has investigated how these methods affect the antioxidant characteristics and phenolic levels in white button mushrooms. Results show that microwave-vacuum drying leads to increased phenolic levels and improved antioxidant activity [8]. A different investigation evaluated how various drying methods—specifically hot air, vacuum, microwave, and microwave-vacuum—impact the essential characteristics and volatile compounds of whole *Lentinula edodes*. The results showed that the microwave-vacuum drying method maintained high levels of flavor-active amino acids while improving nutrient retention and overall quality indicators [22]. Current research on *F. luteovirens* primarily emphasizes its active ingredients, clinical applications, and classification [23]. However, there is little literature on the comprehensive evaluation of the effects of different drying methods on *F. luteovirens* based on metabolomics methods combined with physiological and biochemical indicators [24].

Metabolomics is a branch of omics research that provides valuable insights into the complex composition of biological samples [5,25,26]. The species *F. luteovirens* is rich in polysaccharides, sterols, lipids, proteins, and other metabolites, all of which are of significant interest for extensive scientific exploration. The present research investigates how different drying methods influence the morphological features, microstructure, antioxidant properties, and metabolite profiles of *F. luteovirens* [27]. The composition of both primary and secondary metabolites was analyzed using untargeted metabolomics approaches. Furthermore, bioactive components associated with antioxidant activity were identified through comparative studies, providing significant revelation for the advancement and application of *F. luteovirens*.

## 2. Materials and Methods

### 2.1. Materials

*F. Luteovirens* was purchased from Qinghai Baohuitang Biotechnology Co., Ltd., located in Xining, Qinghai Province, China. Uniformly sized mushrooms, including both cap and stipe, as well as those of consistent maturity, were selected as test materials. Fresh samples of *F. luteovirens* were specifically collected in July 2024 from the Haibei Tibetan Autonomous Prefecture (E95.41°, N33.835°; altitude: 4187.6 m), Qinghai Province, China. All fresh samples were stored in ultracold tanks at −80 °C (Haier Company, Qingdao, China) until they were processed using various drying methods for metabolomics analysis and antioxidant activity determination. Three different drying methods were employed to dry 100 g of fresh *F. luteovirens* (CK): vacuum freeze-drying (DG), oven-drying (HG), and natural air-drying (YG). For vacuum freeze-drying (DG), 100 g of fresh *F. luteovirens* was spread on a tray and then placed in a vacuum freeze-dryer (Pilot 5-8S, Beijing Blocool Laboratory Instrument Co., Ltd., located in Beijing, China) for pre-freezing at −35 °C for 3 h. During this procedure, the cold trap’s temperature dropped to −50 °C, while the vacuum pressure remained consistently between 10 and 40 Pa. Following this, the samples underwent freeze-drying under low-temperature and high-vacuum conditions for a duration of 25 h until a stable weight was reached. In the case of oven-drying (HG), 100 g of fresh *F. luteovirens* was evenly distributed on a tray within a thermostatic electric desiccator (PH030A, Shanghai Yiheng Science and Technology Co., Ltd., located in Shanghai, China) set to a temperature of 60 °C, where hot air was used to maintain the desired temperature. The samples were subjected to the drying process until they attained a stable weight. In the case of air-drying (YG), 100 g of freshly collected *F. luteovirens* was laid out evenly in an air-drying tray, which was then situated in a bright and well-ventilated location exposed to full sunlight. The temperature of the surrounding environment was regulated to remain between 20 and 25 °C, while the relative humidity was kept consistent at 50–65% until the samples reached a steady weight.

### 2.2. Morphological Characterization and Microstructural Analysis

The morphology and coloration of *F. luteovirens* are critical factors in assessing its freshness, identifying undesirable changes, and determining its maturity. This study relied on visual, olfactory, and tactile senses to evaluate the appearance, morphology, color, odor, and hardness of *F. luteovirens*. A comprehensive examination of these sensory traits can serve as a timely and accurate test of the quality of *F. luteovirens* and whether anomalies are present, and the method is both intuitive and straightforward.

The use of scanning electron microscopy (SEM) serves as an effective method for performing ultrastructural evaluations of biological specimens at their surfaces. In this study, a scanning electron microscope (SEM, Hitachi SU8100, Tokyo, Japan) was used to examine the microstructure of fresh and dried *F. luteovirens* samples. The SEM analyses were conducted at magnifications of 150×, 500×, and 1500×, utilizing a voltage of 5.0 kV. Each sample group included three replicates, and the data were processed with IBM SPSS Statistics 26.0 software, while Origin 2022 software facilitated the data visualization. Results from all experiments were expressed as the mean ± standard error (SEM).

### 2.3. Determination of Antioxidant Activity

In this study, the kit utilized was obtained from Suzhou Keming Biotechnology Co., Ltd., which is based in Suzhou, China. The test method was performed according to the instructions provided with the kit and the relevant literature [28,29].

The scavenging capacity of the 2,2-Diphenyl-1-picrylhydrazyl radical (DPPH) was assessed using a total antioxidant capacity assay kit. Approximately 0.1 g of the sample was weighed, and 1 mL of extracting solvent ethanol was added for homogenization in an ice bath. Subsequently, the mixture was centrifuged at 10,000× *g* for 10 min at 4 degrees Celsius, after which the supernatant was collected for measurement. In a blank tube, 50 μL of extract and 950 μL of DPPH solution were combined. In another tube, 50 μL of the sample solution and 950 μL of DPPH solution were added. The mixtures were thoroughly mixed and allowed to react at room temperature in the dark for 20 min. The absorbance of the reaction solution was measured at 515 nm, and the results were calculated using the formula provided below.

DPPH radical scavenging ability (%) = [(A0 − A1)/A0] × 100%

where A0 is the blank value and A1 is the measured value.

The determination of hydroxyl radical (•OH) scavenging capacity involved taking 0.1 g of the sample and adding 1 mL of distilled water to homogenize it in an ice bath. The mixture was then centrifuged at 10,000× *g* and 4 °C for 10 min, after which the supernatant was collected for measurement. In the EP blank tube, 150 μL of the salicylic acid–ethanol solution, 900 µL of distilled water, and 300 µL of H_2_O_2_ solution were added. In the EP measuring tube, 150 µL of the salicylic acid–ethanol solution, 150 µL of FeSO_4_·7H_2_O, 450 µL of distilled water, 300 µL of 4% H_2_O_2_ solution, and 300 µL of the sample solution were combined. The mixture was thoroughly mixed and incubated at 37 °C for 20 min, after which 1 mL was taken to determine the absorbance at 510 nm using a glass cuvette. The results were subsequently calculated using the formula provided below.

●OH radical scavenging ability (%) = [(A0 − A2)/A0 − A1] × 100%

where A0 is the control value, A1 is the blank value, and A2 is the measured value.

The determination of superoxide anion radical scavenging capacity (O_2_^●−^) involves weighing 0.1 g of the sample and adding 1 mL of extracting solvent ethanol to obtain a homogenate in an ice bath. The mixture is then centrifuged at 10,000× *g* for 20 min at 4 °C to obtain the supernatant. To the empty tube, 40 µL of Tris-HCl solution, 100 µL of distilled water, 160 µL of ammonium persulfate solution, 200 µL of hydroxylamine hydrochloride solution, 200 µL of p-aminobenzenesulfonic acid solution, and 200 µL of alpha-naphthylamine acetic acid solution are added. For the sample tubes, 40 μL of Tris-HCl solution, 100 μL of sample solution, 160 μL of ammonium persulfate solution, 200 μL of hydroxylamine hydrochloride solution, 200 µL of p-amino benzene sulfonic acid–acetic acid solution, and 200 µL of alpha-naphthylamine acetic acid solution are combined. The prepared blank tube and the determination tube solution are thoroughly mixed and incubated in a water bath at 37 °C for 20 min. Subsequently, the sample solution is transferred to a 1 mL glass cuvette, and the absorbance is measured at 530 nm. The results are calculated according to the formula provided below.

O_2_^●−^ radical scavenging ability (%) = [(A0 − A1)/A0] × 100%

where A0 is the blank value and A1 is the measured value.

The procedure for determining Ferric Reducing Antioxidant Power (FRAP) was performed as outlined: A sample weighing 0.1 g was measured and mixed with 1 mL of extracting solvent ethanol for homogenization within an ice bath. Following this, the mixture underwent centrifugation at 10,000× *g* and 4 °C for a duration of 10 min, after which the supernatant was collected (LC-400 model centrifuge, Shanghai Yiheng Scientific Instrument Co., Ltd., located in Shanghai, China). Afterward, a necessary mixed solution was prepared, comprising anhydrous sodium acetate, glacial acetic acid, 2,4,6-tris(2-pyridyl)-s-triazine (TPTZ) solution, and FeCl3·6H_2_O solution. A blank tube was prepared by combining 50 µL of the anhydrous sodium acetate and glacial acetic acid mixture with 950 µL of the mixed solution. To each measurement tube, 50 µL of the sample solution and 950 µL of the mixed solution were added. It was ensured that all prepared solutions were mixed thoroughly, then they were allowed to react at room temperature for 20 min, and subsequently, the absorbance at a wavelength of 593 nm was measured. Calculations will be made using the formulas and standard curves provided below.

Labeled curve: A0 = 2.4832x + 0.0134 R^2^ = 0.9996

where x is the Trolox concentration and A0 is the absorbance value difference.

FRAP radical scavenging ability (µmol Trolox/g) = 0.4027 × [(A0 − 0.0134/W]

where A0 is the absorbance value difference and W is the sample mass (g).

### 2.4. Untargeted Metabolomics Analysis by LC-MS/MS

#### 2.4.1. Sample Preparation and Metabolites Extraction

Preparation of samples for untargeted metabolomics was performed following the manufacturer’s guidelines. For each group, six replicates were created. The quality control (QC) sample was generated by combining equal volumes of the supernatants derived from CK, DG, YG, and HG *F. luteovirens*. After a gradual thawing at 4 °C, 100 μL of each sample was taken, to which 400 μL of a chilled methanol/acetonitrile mixture (1:1, *v*/*v*) was incorporated. The resulting solution was vortexed and kept at −20 °C for 30 min. Then, the samples underwent centrifugation at 14,000× *g* at 4 °C for a duration of 20 min, and the supernatants were subsequently harvested and dried under vacuum conditions. To re-dissolve the samples before mass spectrometry (MS) analysis, 100 μL of an acetonitrile solution (1:1, *v*/*v* acetonitrile–water) was introduced. Following this, the samples were centrifuged once more at 14,000× *g* for 15 min at 4 °C, allowing for the supernatants to be collected for further analysis. The QC sample was also prepared by mixing equal portions of the supernatants from CK, DG, HG, and YG *F. luteovirens*.

#### 2.4.2. LC-MS/MS and Mass Spectrum Condition

The analysis of untargeted metabolomics was performed based on earlier established reporting methods with slight alterations [30,31]. Briefly, an Agilent 1290 Infinity LC ultra-high-performance liquid chromatography (UHPLC) HILIC column was employed to separate the samples. The mobile phase was made up of two compositions: composition A, which included water with 25 mM of ammonium acetate and 25 mM of ammonia, and composition B, consisting of acetonitrile. The gradient elution followed this program: from 0 to 0.5 min, composition B was at 95%; between 0.5 and 7 min, B linearly decreased from 95% to 65%; from 7 to 8 min, B continued to decrease linearly from 65% to 40%; from 8 to 9 min, B was held constant at 40%; from 9 to 9.1 min, B increased linearly from 40% back to 95%; and from 9.1 to 12 min, B was maintained at 95%. During the analysis, samples were stored in an autosampler at 4 °C. The column was kept at a temperature of 25 °C, with a flow rate set to 0.5 mL/min and an injection volume of 2 μL. To reduce the impact of variations in the detection signal from the instrument, samples were analyzed in a randomized sequence. Quality control (QC) samples were incorporated into the queue to assess and ensure the system’s stability and the experimental data’s reliability.

A mass spectrometer, the AB Triple TOF 6600 (Shanghai SCIEX Analytical Instruments Trading Co., Ltd., located in Shanghai, China), was utilized to gather primary and secondary spectra from the samples. Following hydrophilic interaction liquid chromatography (HILIC) separation, the conditions for the electrospray ionization (ESI) source were set as follows: Ion Source Gas 1 (Gas1) at 60, Ion Source Gas 2 (Gas2) also at 60, curtain gas (CUR) at 30, a source temperature of 600 °C, ion spray voltage, with the TOF MS scan *m*/*z* range spanning from 60 to 1000 Da, and the product ion scan *m*/*z* range from 25 to 1000 Da. The accumulation time for the TOF MS scan was 0.20 s per spectrum, while the product ion scan accumulation time was 0.05 s per spectrum. Secondary mass spectra were acquired through information-dependent acquisition (IDA) in high-sensitivity mode, with parameters set as follows: declustering potential (DP) at ±60 V for both positive and negative ion modes and a collision energy of 35 ± 15 eV. The IDA parameters included the exclusion of isotopes within 4 Da and the monitoring of 10 candidate ions during each cycle.

The conversion of raw data into MzML format was accomplished with ProteoWizard, followed by the utilization of the XCMS software for tasks such as peak alignment, retention time correction, and extraction of peak areas. Initially, the completeness of the extracted data from XCMS was evaluated, leading to the exclusion of metabolites exhibiting more than 50% missing values within a group from further analysis. Missing data were addressed through K-nearest neighbors (KNN) imputation, while extreme values were eliminated. Ultimately, normalization of the data to the total peak area was performed, facilitating parallel comparisons among samples and metabolites.

#### 2.4.3. Data Processing and Statistical Analysis

Multivariate statistical analysis is a branch that has evolved from classical statistics and serves as a comprehensive method of analysis. In this study, we use principal component analysis (PCA) and orthogonal projection to perform discriminant analysis of latent structures using two methods: unsupervised dimensionality reduction and supervised dimensionality reduction. This approach included all samples, as well as quality control (QC) samples, to elucidate the differences in total metabolism and the degree of variability among the samples. Additionally, the accumulation patterns of metabolites in *F. luteovirens* under various drying methods were analyzed using hierarchical cluster analysis (HCA).

Differentially accumulated metabolites (DAMs) were identified by evaluating variable weight values (VIP) and a significance level of *p*, with metabolites exhibiting VIP > 1 and *p* < 0.05 designated as differential.

Annotation and categorization of DAMs were conducted using the Kyoto Encyclopedia of Genes and Genomes (KEGG) database, which was accessed on 15 September 2024 (http://www.kegg.jp/kegg/pathway.html). The relationship and significance of DAMs concerning antioxidant activity were assessed through SPSS 26.0 software. Furthermore, additional analysis was performed using ChiPlot (https://www.chilot.online/ (accessed on 24 September 2024)).

## 3. Results

### 3.1. Effect of Different Drying Methods on the Morphological Characteristics of F. luteovirens

**Figure 1:**
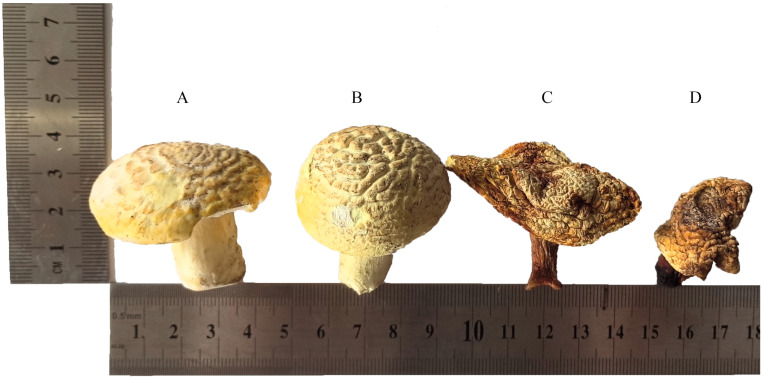
Morphological features of *F. luteovirens* specimens exposed to various drying methods. (**A**) CK: fresh F. *luteovirens*; (**B**) DG: vacuum freeze-drying; (**C**) YG: natural air-drying; (**D**) HG: oven-drying.

Samples of *F. luteovirens* from the CK and DG groups exhibited a golden-yellow color, whereas the YG and HG samples were characterized by a yellowish-brown hue, with the latter demonstrating more pronounced browning. The CK and DG samples had a weak mushroom flavor, while the YG and HG samples had a strong mushroom flavor. In terms of morphology and texture, both YG and HG samples displayed noticeable shrinkage, accompanied by a firmer texture (Figure 1A–D). In conclusion, *F. luteovirens* dried by the DG method had the closest color to that of fresh *F. luteovirens*, with the least browning, and was able to maintain the morphology and flavor of fresh *F. luteovirens* to the greatest extent.

**Figure 2:**
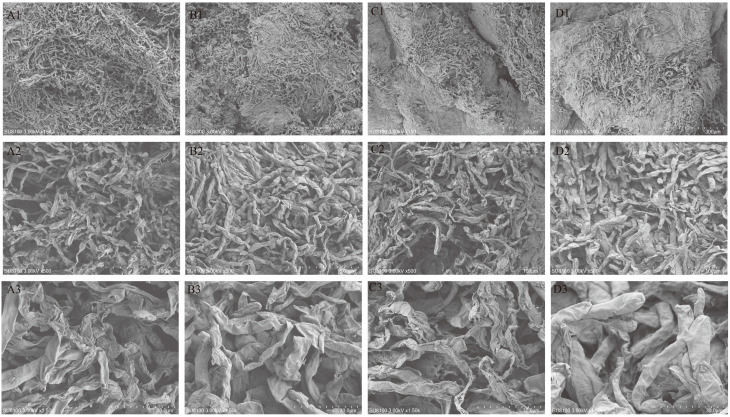
The microstructural characteristics of *F. luteovirens* samples exposed to various drying methods. (**A1**–**A3**) CK; (**B1**–**B3**) DG; (**C1**–**C3**) YG; (**D1**–**D3**) HG. Scale bar: (**A1**–**D1**) at 150× magnification, (**A2**–**D2**) at 500× magnification, and (**A3**–**D3**) at 1500× magnification.

The scanning electron microscope (SEM) serves as an observational method that lies between the transmission electron microscope and the optical microscope [32]. The results obtained from SEM analysis demonstrated the microstructure of fresh and dried samples of *F. luteovirens* (Figure 2A1–D1). The CK sample displayed a full structure characterized by large inter-tissue pores and a three-dimensional look. In contrast, the DG group samples displayed a porous reticulate structure characterized by larger pores, looser organization, and enhanced structural integrity. Notably, pre-freezing at −35 °C resulted in a slower freezing rate, leading to the formation of ice crystals and larger pores. The HG group samples, however, exhibited small pores and dense organization, alongside a collapse phenomenon, which may be attributed to the higher drying temperature that compromised cellular organization. Additionally, the YG group samples demonstrated an uneven distribution of pores, with some areas being less pronounced; the reticulate structure was also less distinct, and the inner portion of the cap comprised numerous swollen tubular mycelia. In summary, various drying methods significantly influenced the micromorphology of *F. luteovirens*.

### 3.2. Antioxidant Activity Assay

**Figure 3:**
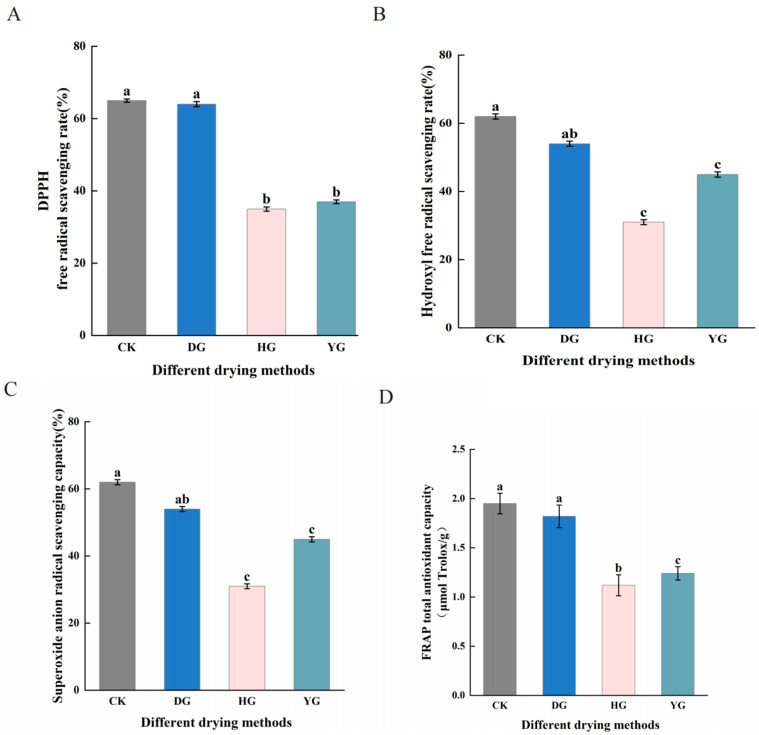
Antioxidant capacity of *F. luteovirens* under various drying methods. (**A**) Scavenging capacity for the 2,2-Diphenyl-1-picrylhydrazyl radical (DPPH); (**B**) scavenging ability against hydroxyl radicals (●OH); (**C**) superoxide anion radical scavenging ability (O_2_^●−^); (**D**) Ferric Reducing Antioxidant Power (FRAP). Letters that differ signify statistically significant variations among groups. Identical letters denote no significant differences between the respective groups (*p* > 0.05), whereas differing letters indicate a significant difference (*p* < 0.05).

Antioxidant activity is the ability to resist oxidative free radicals, which are strong oxidizing species that cause cell aging and disease [33]. As a result, the impact of different drying techniques on the antioxidant activity of *F. luteovirens* was assessed by evaluating the scavenging rates of DPPH, ●OH, and O_2_^●−^ radicals, in addition to determining the overall antioxidant capacity through the FRAP assay (Figure 3). The findings revealed that the samples from the CK group exhibited the highest DPPH radical scavenging rates at 65 ± 0.46%, closely followed by the DG group with 64 ± 0.7%. No significant difference was found between these two groups (*p* < 0.05), whereas the HG group showed the lowest DPPH radical scavenging rate at 35 ± 0.58% (Figure 3A).

The ●OH and O_2_^●−^ radical scavenging rates demonstrated strong consistency across the four sample groups. The scavenging rates for the DG group were second only to those of the CK group, indicating a robust antioxidant capacity (Figure 3B,C). The YG group demonstrated marginally greater scavenging rates compared to the HG group. Additionally, significant differences were observed in total antioxidant capacity (FRAP) among the four groups. The CK group (1.95 µmol Trolox/g) and the DG group (1.82 µmol Trolox/g) did not show a significant difference between them (*p* > 0.05). The HG group exhibited the lowest total antioxidant capacity (FRAP) at 1.12 µmol Trolox/g. In summary, the CK and DG methods demonstrated exceptionally strong antioxidant activity compared to the other two drying methods (Figure 3D).

### 3.3. Metabolite Profiles Analysis of F. luteovirens

**Figure 4:**
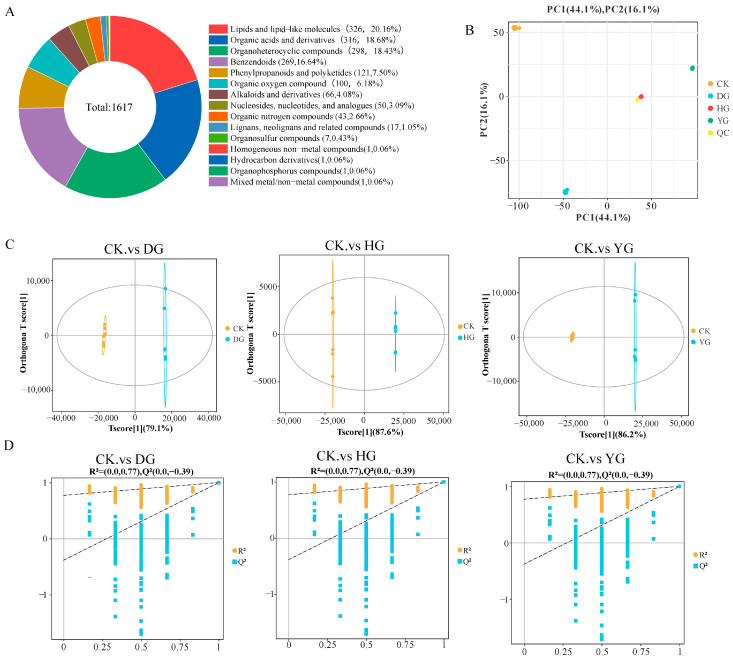
Metabolite classification profile and multivariate statistical evaluation of *F. luteovirens*. (**A**) The classification of metabolites into superclasses was observed in the CK, DG, HG, and YG groups; (**B**) principal component analysis (PCA) was performed on samples from the four groups along with QC quality control samples; (**C**) OPLS-DA score plots for CK, DG, YG, and HG; (**D**) permutation testing for OPLS-DA results of CK, DG, YG, and HG.Note:The two dashed lines represent the regression lines for R2Y and Q2 respectively, Circle for R^2^, Rectangle for Q^2^.

**Figure 5:**
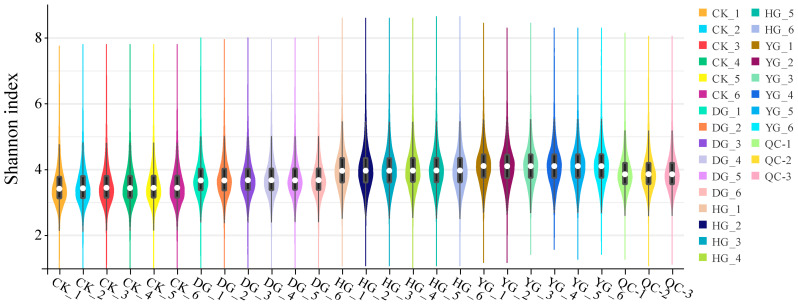
Violin plots of abundance expression of all sample metabolites. Note: The black rectangle indicates the units from the lower quartile to the upper quartile, the white dot indicates the median, the length of the rectangle indicates the degree of dispersion and symmetry of the non-anomalous data, with the longer ones being dispersed and the shorter ones being centralized, and the black lines indicate the confidence intervals, with the upper and lower ends representing the upper and lower limits, respectively.

Metabolomics, which involves analyzing metabolites found in biological specimens, offers an in-depth perspective of the condition of the sample [34]. As a result, metabolomics was utilized to meticulously assess the makeup of these compounds. The base peak chromatograms (BPCs) acquired from both positive and negative ion detection modes were overlaid, revealing a significant overlap that suggested uniform retention times and peak intensities. This observation highlights the stability and reliability of the data collected by the detection system in both detection modes—positive ion detection mode in Figure S1A and negative ion detection mode in Figure S1B [35,36]. A total of 1617 metabolites with relevant classification information were identified in this study (Table S1). These include 326 lipids and lipid-like molecules, 316 organic acids and derivatives, 298 organic heterocyclic compounds, and 269 benzenoid compounds (Figure 4A). To delve deeper into the inherent variations in metabolites among the groups, a principal component analysis (PCA) was performed on the metabolite data. The PCA score plot revealed that the initial two principal components explained 70.3% of the variance (PC1 = 44.1%; PC2 = 16.1%) (Figure 4B). The findings indicated that the six biological replicates belonging to each sample group were tightly clustered, thereby validating the consistency and dependability of the LC-MS technique. The OPLS-DA evaluation model’s parameters encompass R^2^X, R^2^Y, and Q^2^; R^2^X and R^2^Y represent the explanatory capacity of the X and Y matrices within the constructed model, whereas Q^2^ signifies the model’s capability for prediction. The stability and reliability of the model are considered higher when the three indices approach 1 [37,38]. OPLS-DA analysis results demonstrated that, for each model, the two sample groups were contained within a 95% confidence ellipse, with R^2^Y exceeding 0.5 and Q^2^ surpassing 0.5, which implies that the model is both stable and trustworthy (Figure 4C). The outcomes of the permutation test involving 200 randomly ordered plots displayed a gradual decline in R^2^ and Q^2^ values. Additionally, the Q2 intercept obtained from the permutation test for all three sample groups was −0.39, suggesting that the original model did not suffer from overfitting and that the differentiation of metabolites between the groups was statistically significant (Figure 4D). Violin plots for all samples show the distribution of multiple subgroups, with stable and reliable data between groups and no anomalous data (Figure 5).

### 3.4. Correlation Between Antioxidant Capacity and Metabolites

**Figure 6:**
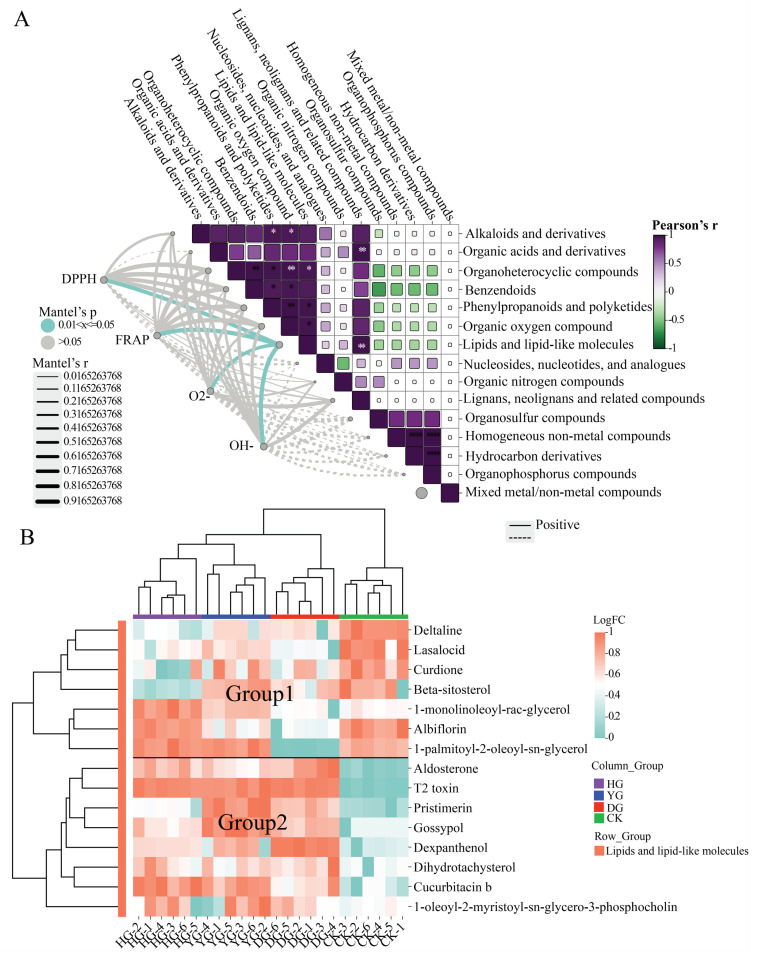
(**A**) The relationship between the antioxidant capacity and the varied metabolites of *F. luteovirens* subjected to various drying techniques (* *p* < 0.05; ** *p* < 0.01, and *** *p* < 0.001); (**B**) heatmap illustrating the pattern of accumulation of lipids and lipid-like substances in *F. luteovirens* sourced from three distinct drying methods.

The relationship between the antioxidant capacity of *F. luteovirens* and several superclass metabolites subjected to various drying techniques was examined (Figure 6A). Out of the 15 compound superclasses investigated, significant correlations were found between lipids and lipid molecules and the scavenging rates of DPPH, ●OH, and O_2_^●−^ free radicals, along with the total antioxidant capacity measured by FRAP. The results showed that the antioxidant capacity varied under different drying methods, which was related to the content of portion compounds. Figure 6B comprehensively analyzed the effects of different drying methods on the first 15 lipids and lipid-like molecules metabolites and obtained a heatmap. The four groups of samples were clustered into two classes: the HG, YG, and DG groups were clustered into one class, and the CK group was clustered into a separate class on its own. Closer inspection revealed that the YG and HG groups were divided into one category in one step, and the DG group clustered into one category. The lipid and lipid-like molecules of *F. luteovirens* under different drying methods showed completely different accumulation patterns from CK samples. These results suggest that drying methods significantly affect the composition of *F. luteovirens* metabolites and that this difference may be caused by differences in treatment temperature.

### 3.5. Screening of Differentially Accumulated Metabolites (DAMs)

**Figure 7:**
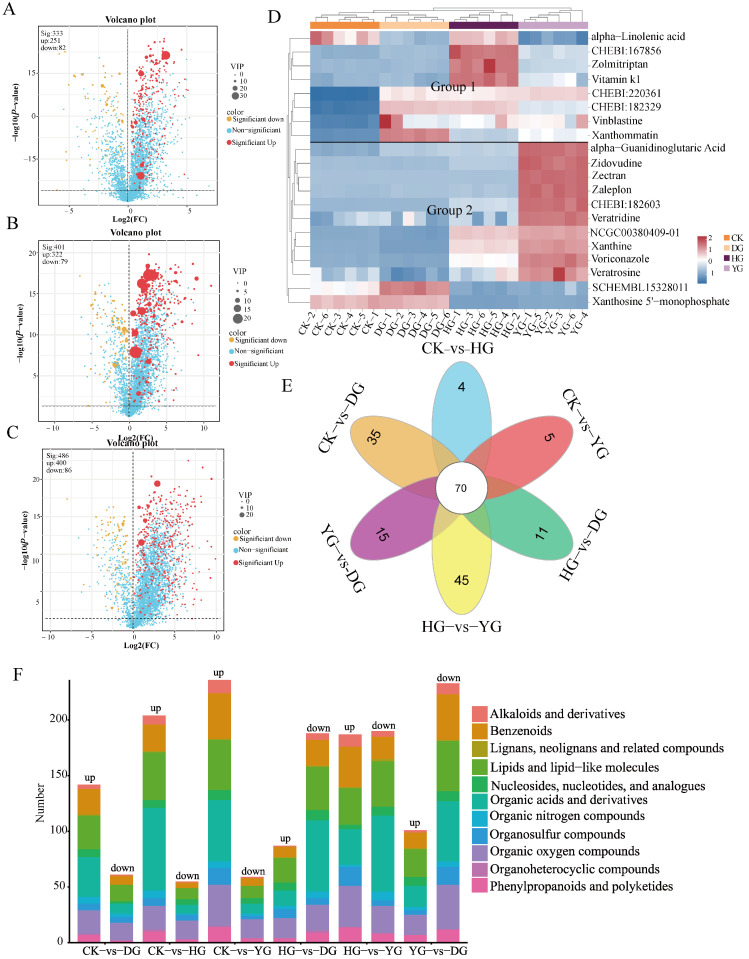
Screening for differentially accumulated metabolites in *F. luteovirens*. (**A**) Volcano plots of CK vs. DG; (**B**) volcano plots of CK vs. HG; (**C**) volcano plots of CK vs. YG; (**D**) HCA of the top 20 DAMs in all samples; (**E**) Venn diagram illustrating both overlapping and distinct metabolites among the groups; (**F**) superclass categorization of DAMs in pairwise comparisons.

The screening criteria for differential accumulated metabolites in each pairwise comparison were a *p*-value < 0.05 and VIP > 1. The DAMs were illustrated using volcano plots (Figure 7A–C) and Venn diagrams (Figure 7E). To visually demonstrate the effects of various drying methods on the metabolites of *F. luteovirens*, the expression of significantly different cumulative metabolites among the top 20 was hierarchically clustered based on VIP values. In the comparison of CK vs. DG, 383 significant differential accumulated metabolites (DAMs) were identified, comprising 251 upregulated and 82 downregulated metabolites (Figure 7A). In the CK vs. HG comparison, 401 significant DAMs were identified, with 322 upregulated and 79 downregulated (Figure 7B). Finally, in the CK vs. YG comparison, 486 differential metabolites were identified, including 400 upregulated and 86 downregulated (Figure 7C). The identified compounds primarily consisted of lipid and lipid-like molecules, organic acids and derivatives, and organic heterocyclic compounds. These metabolites may be associated with the varying antioxidant activities observed (Figure S2).

The results from the HCA revealed that the metabolites with varying accumulation levels clustered into two separate groups. Group 1 included eight metabolites that exhibited a general trend of downregulation, whereas Group 2 comprised twelve metabolites that demonstrated a general trend of upregulation (Figure 7D). Both the HG and YG groups showed large fluctuations in metabolites, and the consistent trend of metabolites in the CK and DG groups indirectly indicated that the DG method helped to maintain the stability of *F. luteovirens* metabolites. A substantial number of differentially accumulated metabolites were classified as lipids and lipid-like molecules, organic heterocyclic compounds, organic acids and derivatives, as well as nucleosides, nucleotides, and their analogues. Notably, nucleosides, nucleotides, and their analogues were significantly upregulated in samples from the CK and DG groups, exhibiting an accumulation pattern that contrasted with samples from the HG and YG groups. The results of the Venn diagram showed that there were 70 metabolites in the six comparison groups, and all the inter-group comparisons contained specific metabolites, and the HG vs. YG group had the most specific metabolites (Figure 7E).

The results of the superclass categorization of differentially accumulated metabolites (DAMs) in intergroup comparisons revealed that lipids and lipid molecules, organic acids and derivatives, and benzenoids were significantly upregulated, particularly evident in the CK compared to the HG and YG groups (Figure 7F).

### 3.6. KEGG Pathway Analysis

**Figure 8:**
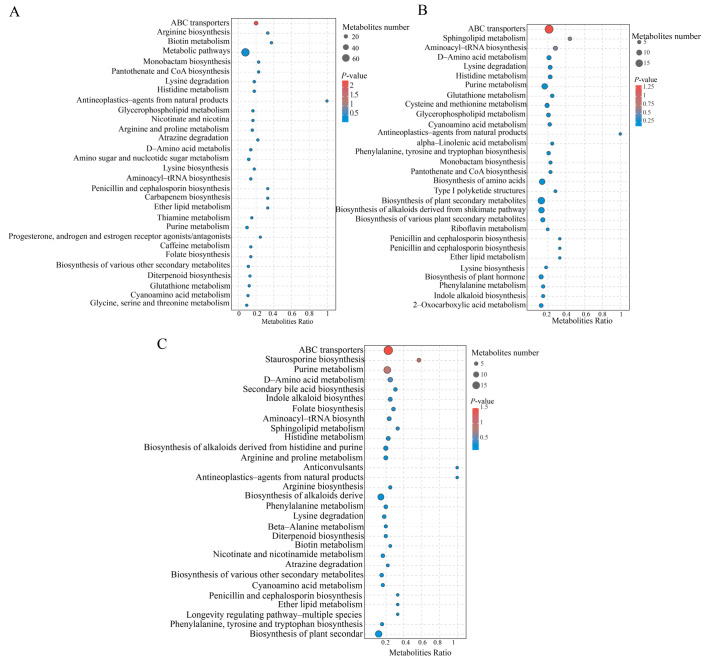
KEGG enrichment analysis. (**A**) Comparison of CK versus DG; (**B**) comparison of CK versus HG; (**C**) comparison of CK versus YG. The circle size in this pathway indicates the quantity; larger circles correspond to greater quantities. Additionally, the color of the circles reflects the *p*-value, with a deeper red indicating a higher *p*-value.

The Kyoto Encyclopedia of Genes and Genomes (KEGG) database (https://www. Gen-ome.jp/kegg/ (accessed on 15 September 2024)) was used to understand metabolism in differential samples through pathway enrichment analysis of differential metabolites. This is the primary resource on mechanisms of pathway change. We annotated the differentially abundant metabolites (DAMs) in the CK vs. DG, CK vs. HG, and CK vs. YG comparison groups, categorizing them into various metabolic pathways. Furthermore, the DAMs in the CK vs. DG, CK vs. HG, and CK vs. YG groups were associated with 67, 74, and 72 pathways, respectively, with the primary pathways illustrated in bubble diagrams (Figure 8A–C).

**Figure 9:**
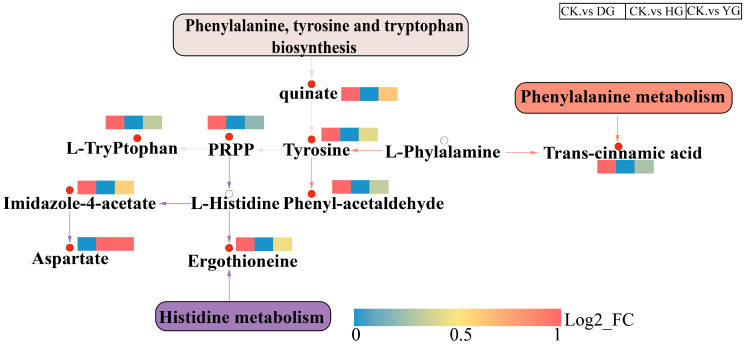
Overview of metabolic pathways, identification of potentially important regulatory metabolites, and comparison between groups of flavobacteria with different drying methods. Note: Small red circles represent metabolites with significant upregulation, while hollow circles denote metabolites that were not detected. Additionally, small red rectangles indicate noteworthy increases in metabolites across groups, and small blue rectangles highlight significant decreases in metabolites between the groups.

An overview of changes in metabolic regulation was provided through the analysis of metabolic pathways related to the most significant differential metabolites, utilizing KEGG and enrichment maps (Figure 9). A key discovery was the marked reduction in metabolic pathways linked to amino acid biosynthesis, especially concerning the production of phenylalanine, tyrosine, and tryptophan, along with the metabolism of phenylalanine and histidine when comparing the CK versus HG and CK versus YG groups. Moreover, there was a notable downregulation of metabolic pathways in the comparison between the CK and DG groups. In the biosynthesis pathways of phenylalanine, tyrosine, and tryptophan, alongside the metabolism of phenylalanine and histidine, metabolites including quinate, tyrosine, and L-tryptophan showed an increase in their levels. Consequently, it is hypothesized that factors related to temperature and duration of various drying methods could impact the synthesis and metabolism of phenylalanine.

## 4. Discussion

Fresh *F. luteovirens* samples were processed using various drying methods, which resulted in alterations to their color, texture, microstructure, and metabolite profiles. This research utilized the LC-MS/MS metabolomics method to identify a total of 1617 metabolites from four groups of samples, which were later categorized into 15 unique superclasses. The complexity and diversity of the metabolite composition of *F. luteovirens* are evident. A pairwise comparison of the four sample sets indicated that the predominant metabolite types, whether CK, DG, YG, or HG, were largely present in categories like lipids and amino acids. This observation aligns with the findings from previous analyses of *F. luteovirens* metabolites reported in related studies [39,40]. Nonetheless, an in-depth analysis of additional data showed considerable discrepancies in both the quantity and composition of numerous metabolites among the groups, indicating that the drying technique significantly influences metabolite development.

Analysis of the data indicated that the HG and YG treatments notably improved the metabolic pathways linked to amino acids when compared to the DG group. However, the levels of several metabolites involved in amino acid biosynthesis were reduced. Notably, L-tyrosine, L-histidine, and L-phenylalanine emerged as the primary components of desirable amino acids, predominantly found in the HG and YG groups. This finding aligns with previous descriptions of the morphological characteristics. Furthermore, the HG and YG methods significantly influenced the odor of *F. luteovirens*. This phenomenon may be attributed to the higher total content of sulfur-containing flavor compounds and free amino acids in the *F. luteovirens* products dried using the HG and YG methods, which resulted in a pronounced mushroom flavor. In contrast, the *F. luteovirens* products dried by the DG method exhibited the greatest variety of sulfur-containing compounds, but presented a milder mushroom flavor [41,42]. Drying temperature is a crucial factor that influences the drying rate, which in turn affects the wrinkling of *F. luteovirens* [43]. Under low-temperature conditions, the moisture diffusion rate from the interior to the exterior of *F. luteovirens* matches the evaporation rate from the surface, which hinders the development of a steep moisture gradient. Consequently, uniform contraction does not occur until the final stages of drying, resulting in minimal volumetric crumpling. Conversely, at higher drying temperatures, a more pronounced moisture gradient develops throughout *F. luteovirens*, leading to increased thermal and shrinkage stresses, which ultimately results in significant volume shrinkage [44,45].

This may explain why the DG method is effective in preserving the original morphology of *F. luteovirens*. The drying process involves mass and heat transfer mechanisms that can lead to deformation and increased internal tension in *F. luteovirens*, ultimately damaging its internal organization and altering its microstructure [46]. In contrast, the *F. luteovirens* samples treated with the DG method remained structurally intact, exhibiting numerous and uniform pores between tissues with a three-dimensional appearance. Conversely, the dried *F. luteovirens* samples obtained through the HG and YG methods displayed an uneven distribution of pores and significant internal particle linkages. This discrepancy may be attributed to the DG method’s rapid freezing and vacuum conditions, which effectively preserve the original microstructure of the material’s tissue cells. In contrast, the HG and YG methods, characterized by slower drying rates and prolonged drying times, result in greater destruction of the tissue structure, leading to non-uniform pore sizes [47,48].

*F. luteovirens* exhibits significant pharmacological properties, mainly owing to its ability to act as an antioxidant. This research assessed the antioxidant potential of *F. luteovirens* through four different indices: DPPH, OH, O_2_^●−^ radical scavenging, and the FRAP total antioxidant capacity, all evaluated under a range of drying methods. The findings suggested that the DG samples showed a greater antioxidant capacity in comparison to all other dried samples. The poor antioxidant capacity of the HG and YG methods may be attributed to the prolonged heating technique and oxygen exposure time during the drying process, which usually hastens the breakdown of active components linked to antioxidant activity. In contrast, the low temperature and pressure involved in the DG method inhibit many compounds from interacting with oxygen, thus maintaining their elevated antioxidant capabilities. Analysis of correlation indicated a substantial link between lipids and lipid-related molecules in relation to antioxidant capacity. The metabolites of *F. luteovirens*, particularly lipid and lipid-like molecules, tended to increase under different drying conditions. Significantly, the DG technique produced a greater concentration of these lipid and lipid-like compounds than the other two drying approaches, which may explain its superior antioxidant activity.

In this research, we identified 1617 metabolites across 15 different classes, highlighting that the main active compounds in *F. luteovirens* are lipid molecules, organic acids along with their derivatives, and organic heterocyclic compounds. Lipids are essential in influencing the characteristics of mushrooms, such as their flavor, palatability, and nutritional value [49,50]. Notably, lipids and lipid molecules, as one of the most significant superclasses in *F. luteovirens*, exhibited a markedly differential accumulation of metabolites (DAMs). Variations in these metabolites substantially influence the impact of different drying methods on the quality of *F. luteovirens*. Furthermore, different drying methods significantly affected the content of lipid and lipid-like molecules in *F. luteovirens*. Oxidation is a major contributor to food quality degradation, primarily arising from the adverse effects of high temperatures and elevated oxygen levels.

The DG method demonstrates a distinct advantage in preserving the freshness characteristics of *F. luteovirens* compared to other methods, primarily due to its ability to reduce lipid oxidation. This method operates at low temperatures, which maintains the cells in a dormant state and renders the cell wall less susceptible to damage, potentially resulting in a decrease in the release of lipids [51]. Additionally, the DG method minimizes the exposure of *F. luteovirens* to air for extended periods and helps to preserve lipid metabolites or slow down their lipid degradation process to varying degrees. Conversely, the YG method is a relatively gentle drying technique that effectively regulates the moisture content of *F. luteovirens* and concurrently slows down the oxidation rate [52,53]. The results not only lay the groundwork for additional investigations into the pharmacologically active compounds found in *F. luteovirens*, but they also offer important perspectives for the future creation and use of functional foods and medications.

## 5. Conclusions

This research examines the morphological characteristics, antioxidant properties, and metabolite profiles of four sets of *F. luteovirens* samples obtained from CK, DG, HG, and YG. The findings of the study reveal that the DG samples display morphological traits that are strikingly like those observed in the CK samples, suggesting a notable resemblance in their physical structure. Furthermore, these DG samples have been shown to possess significant antioxidant properties, indicating their potential utility in various applications where protection against oxidative stress is beneficial. The metabolite composition of *F. luteovirens* is evidently complex and diverse. Volcano plots from pairwise comparisons reveal that different drying methods significantly alter the metabolite composition of *F. luteovirens*. Notably, the DG method better preserves metabolite stability compared to the other two drying methods, avoiding significant fluctuations. Additionally, the KEGG enrichment analysis revealed 20 distinct pathways, indicating that the metabolism of amino acids could be a crucial metabolic pathway linked to the drying process of *F. luteovirens*. In conclusion, this research emphasizes the significant potential of the DG method for the preservation of *F. luteovirens*, offering a robust theoretical foundation for its advancement and utilization.

## Data Availability

All data used in this study can be found in the manuscript or Supplementary Materials and can also be obtained free of charge from the corresponding author.

## Supplementary Materials

The following supporting information can be downloaded at: https://www.mdpi.com/article/10.3390/jof11010078/s1. Table S1: Detailed list of metabolites detected. Figure S1A,B: A: Total ion chromatograms of NEG; B: total ion chromatograms of POS. Figure S2: Classification of differential metabolites.
