# Supplementary figures and images for: Influence of Drying Methods on the Morphological Features, Microstructural Properties, and Antioxidant Performance of Floccularia luteovirens: A Metabolomic Analysis
Source: J Fungi (Basel). 2025 Jan 19;11(1):78. doi: 10.3390/jof11010078 (PMC11766630; doi:10.3390/jof11010078)

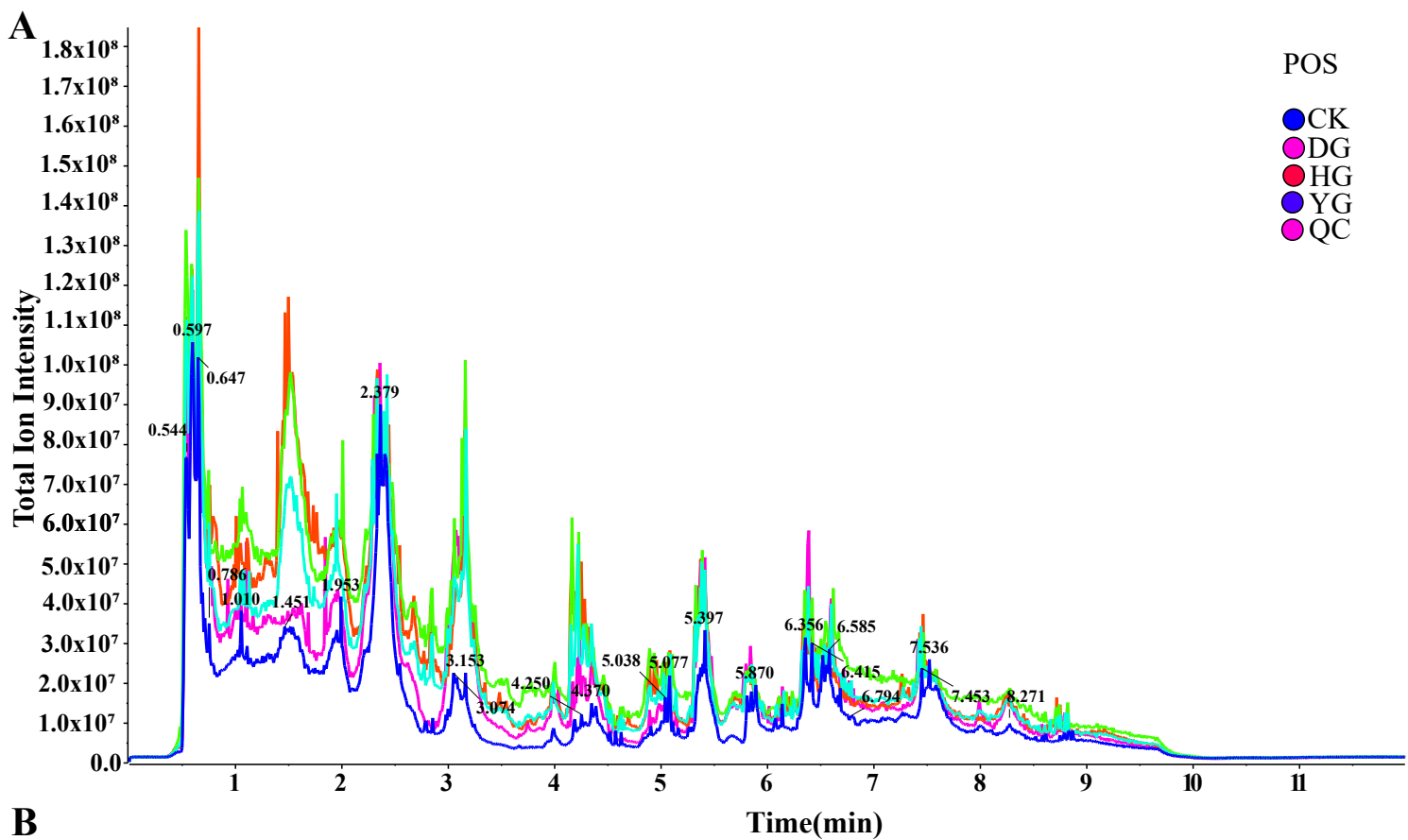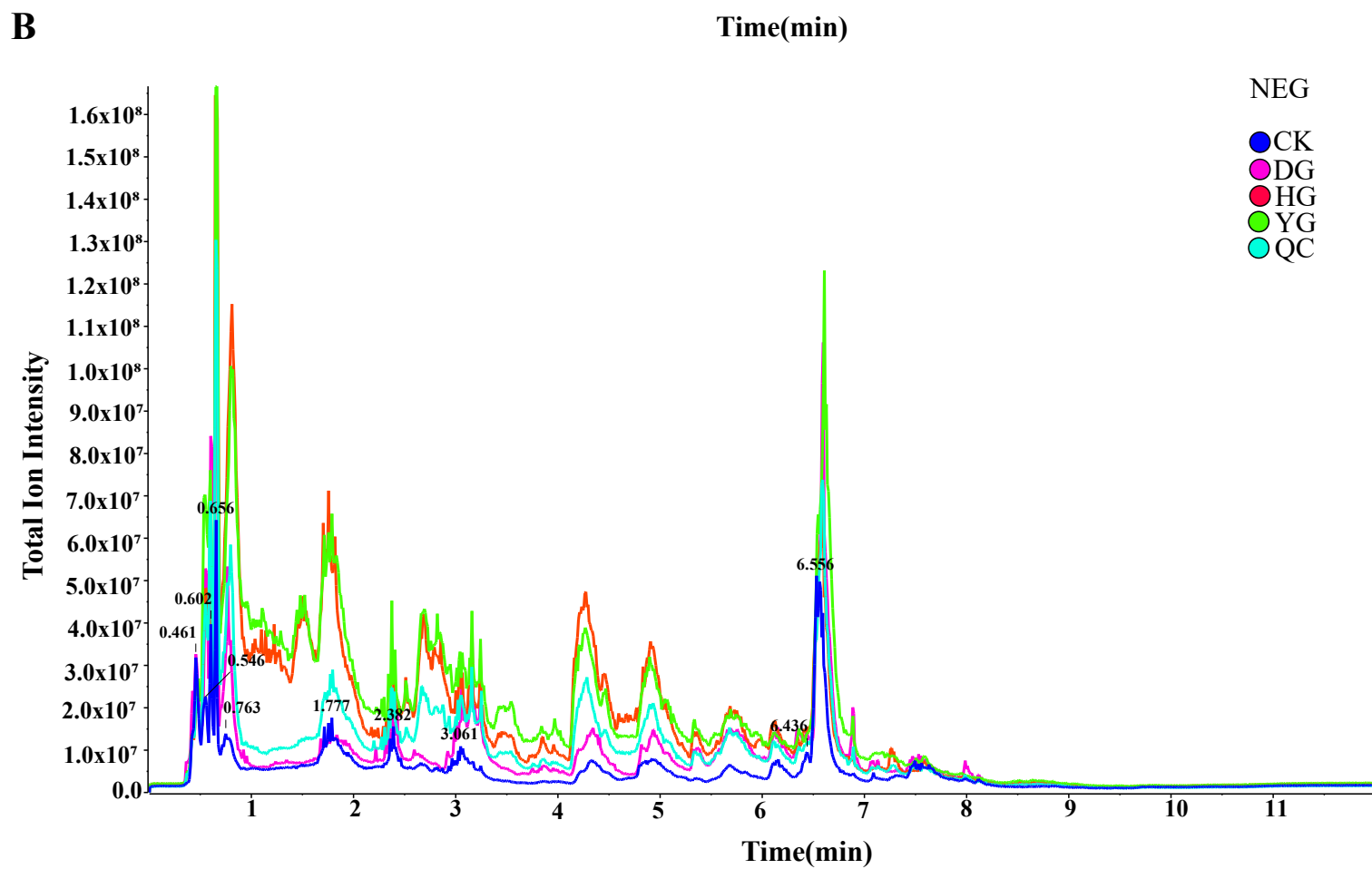

Supplement: Supplementary file 1 [file jof-11-00078-s001.zip › Supplementary material/Figure S1.pdf]

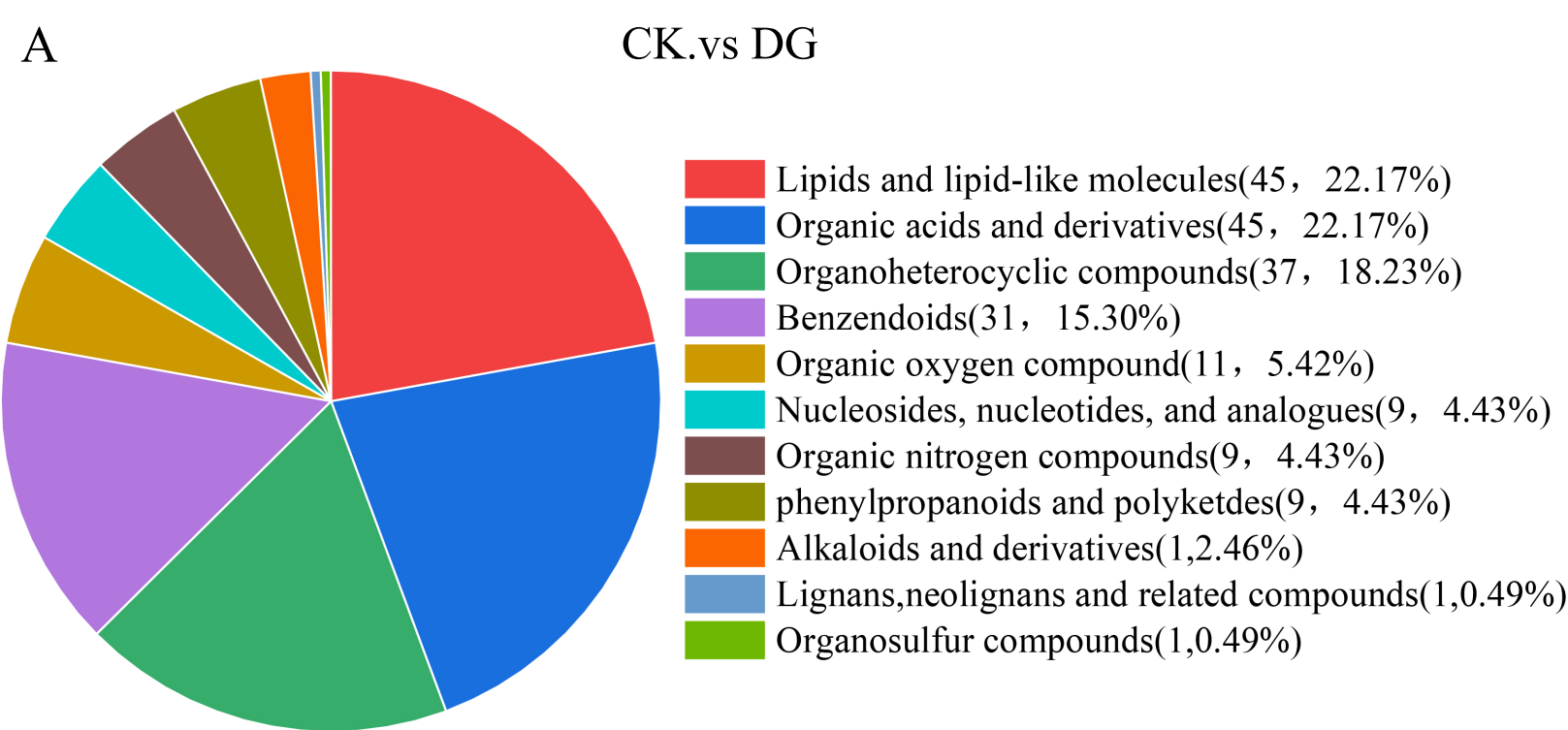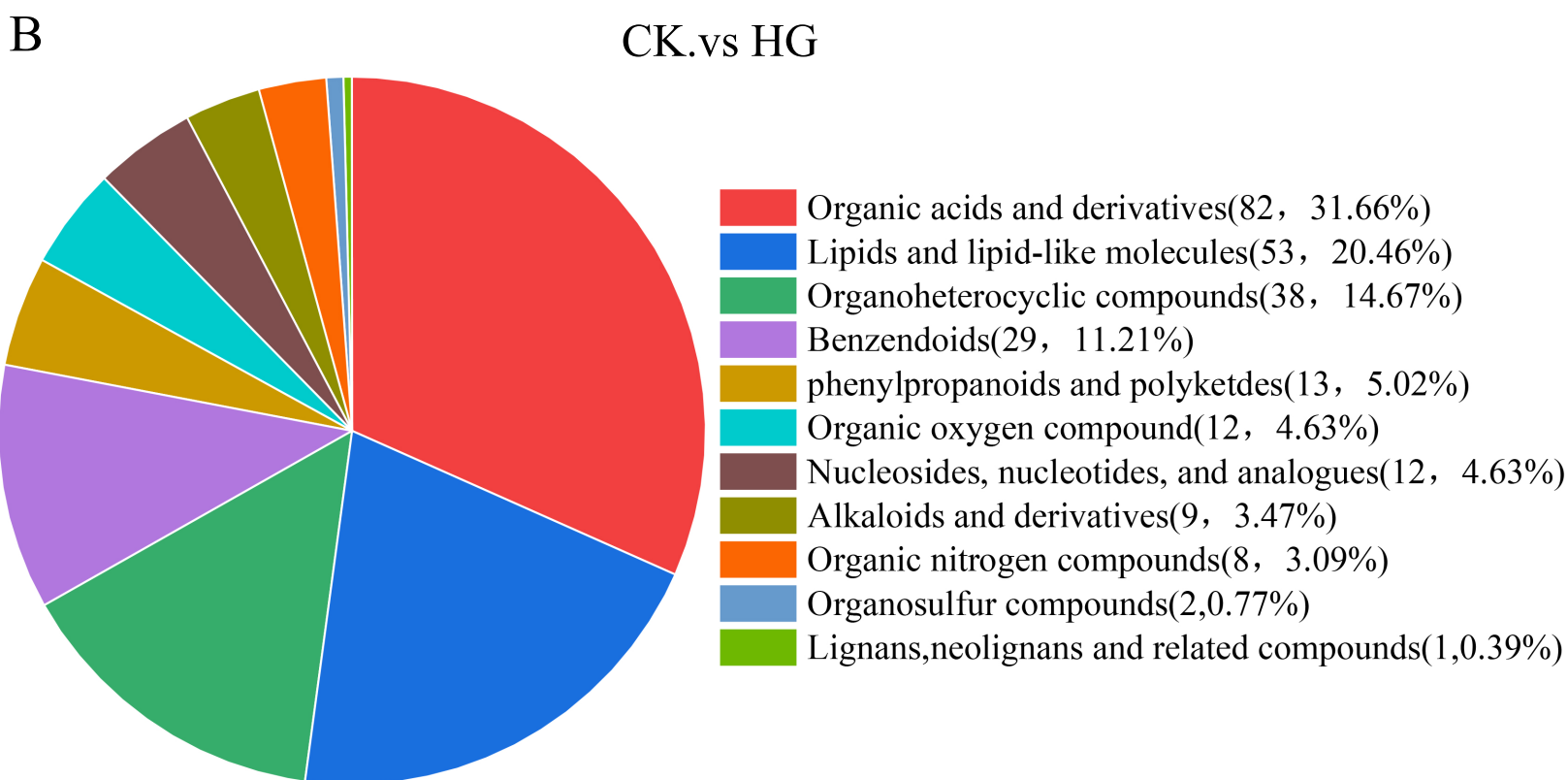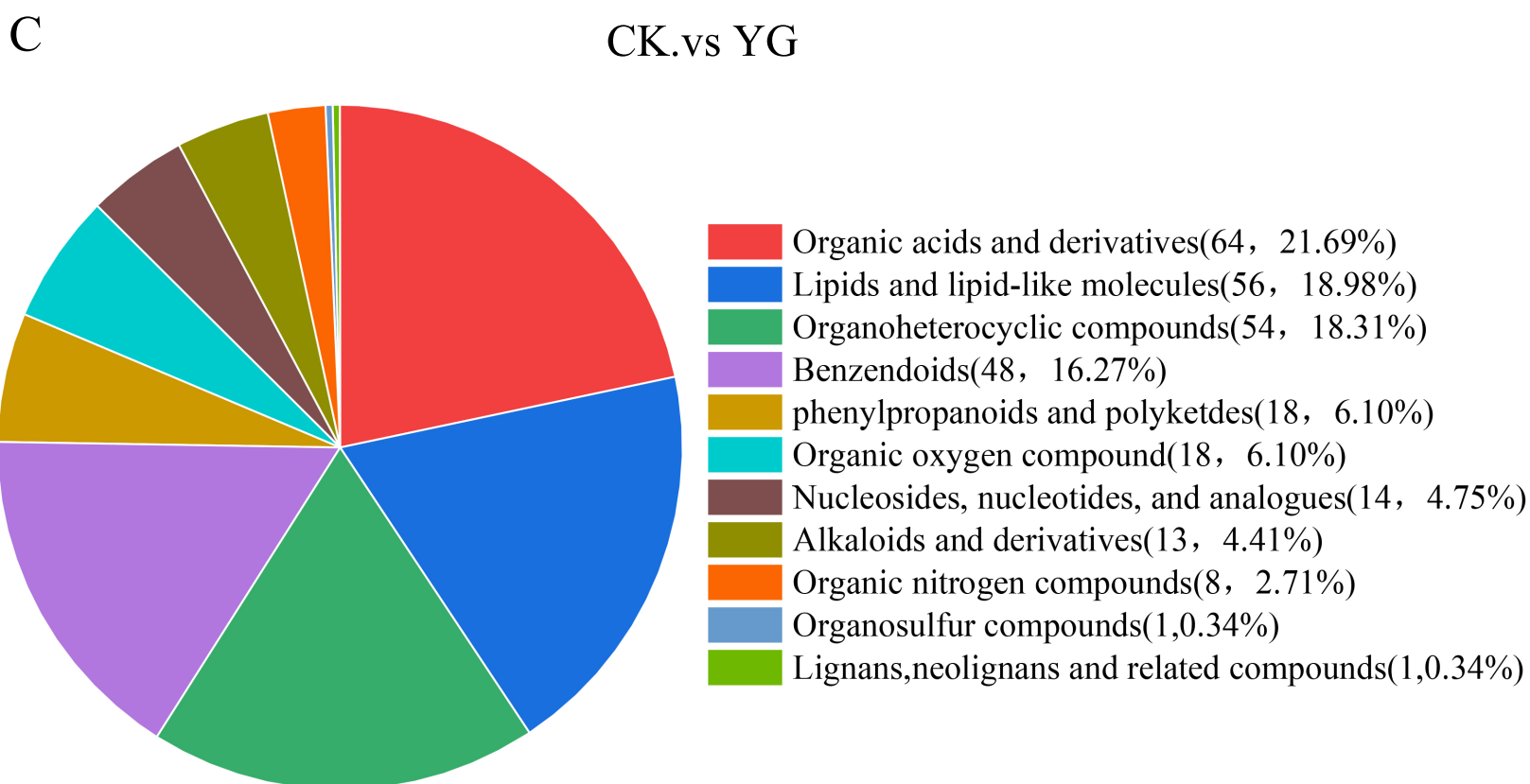

Supplement: Supplementary file 1 [file jof-11-00078-s001.zip › Supplementary material/Figure S2.pdf]
